# Innovation in the U.S. beef value chain: a sustainable beef research roadmap

**DOI:** 10.1093/af/vfag026

**Published:** 2026-06-03

**Authors:** Samantha J Werth, Grace D Vehige, Sara Place, Logan R Thompson, Troy N Rowan, Douglas J Goodwin, Jason E Rowntree, Paige L Stanley, Susan T Metzger, Kevin L Wagner

**Affiliations:** U.S. Roundtable for Sustainable Beef, Centennial, CO 80112; U.S. Roundtable for Sustainable Beef, Centennial, CO 80112; AgNext and Department of Animal Science, Colorado State University, Fort Collins, CO 80523; Department of Animal Sciences and Industries, Kansas State University, Manhattan, KS 66506; Department of Animal Science, University of Tennessee Institute for Agriculture, Knoxville, TN 37996; Department of Large Animal Clinical Sciences, University of Tennessee Institute for Agriculture, Knoxville, TN 37996; Center for Grazinglands and Ranch Management, Texas A&M University, Collage Station, TX 77843; Department of Animal Science, Michigan State University, East Lansing, MI 48824; Department of Soil and Crop Science, Colorado State University, Fort Collins, CO 80523; School of Leadership Studies, Kansas State University, Manhattan, KS 66506; Oklahoma Water Resources Center, Oklahoma State University, Stillwater, OK 74078

**Keywords:** environmental stewardship, greenhouse gas mitigation, measurement and modeling frameworks, producer adoption and practicality, sustainable beef production

Implications

- Coordinated, long-term research efforts are required to provide accurate measurements, scalable tools, and robust data to advance sustainability outcomes across air, land, and water indicators.
- Emerging technologies—including methane-mitigating additives, remote sensing, virtual fencing, genetic selection tools, and continuous monitoring–offer promising pathways for improvement, but their efficacy, scalability, and economic feasibility must be validated across production systems.
- Enhanced modeling frameworks and interoperable data systems are essential to improve environmental benchmarking, inform supply chain reporting, and strengthen producer-level decision-making.
- Producer practicality and adoption are central to achieving sustainability goals, making producer engagement, economic viability, and real-world feasibility critical components of all research and innovation efforts.

## Introduction

The United States is a major global beef supplier, operating a highly diverse production system that spans extensive rangelands and intensive feedyards. Figure 1 provides a conceptual overview of the U.S. beef supply chain, illustrating the major production stages and their connections from cow-calf through feedyard and processing, consistent with established descriptions of the supply chain in the literature (Thoma et al., 2017; Asem-Hiablie et al., 2019; Rotz et al., 2019). While a complex supply chain, representing over 700,000 individual producers and countless supply chain partners, decades of innovation in genetics, nutrition, reproduction, and land management have enabled the industry to improve efficiency and reduce environmental impacts per unit of beef (Capper, 2011; Herrero et al., 2013; Rotz et al., 2019; Klopatek and Oltjen, 2022; Putman et al., 2023; USDA NASS, 2024). Despite this progress, a key challenge remains: how to generate clear, consistent, and actionable evidence of sustainability performance and identify scalable solutions that can be implemented across highly heterogeneous production systems. This is a challenge driven by increasing expectations across stakeholder groups (i.e., producers, packers and processors, retail and food service companies, consumer packaged goods companies, other supply chain partners, and policymakers) for transparent metrics, continuous improvement, and coordinated action.

**Figure 1.**
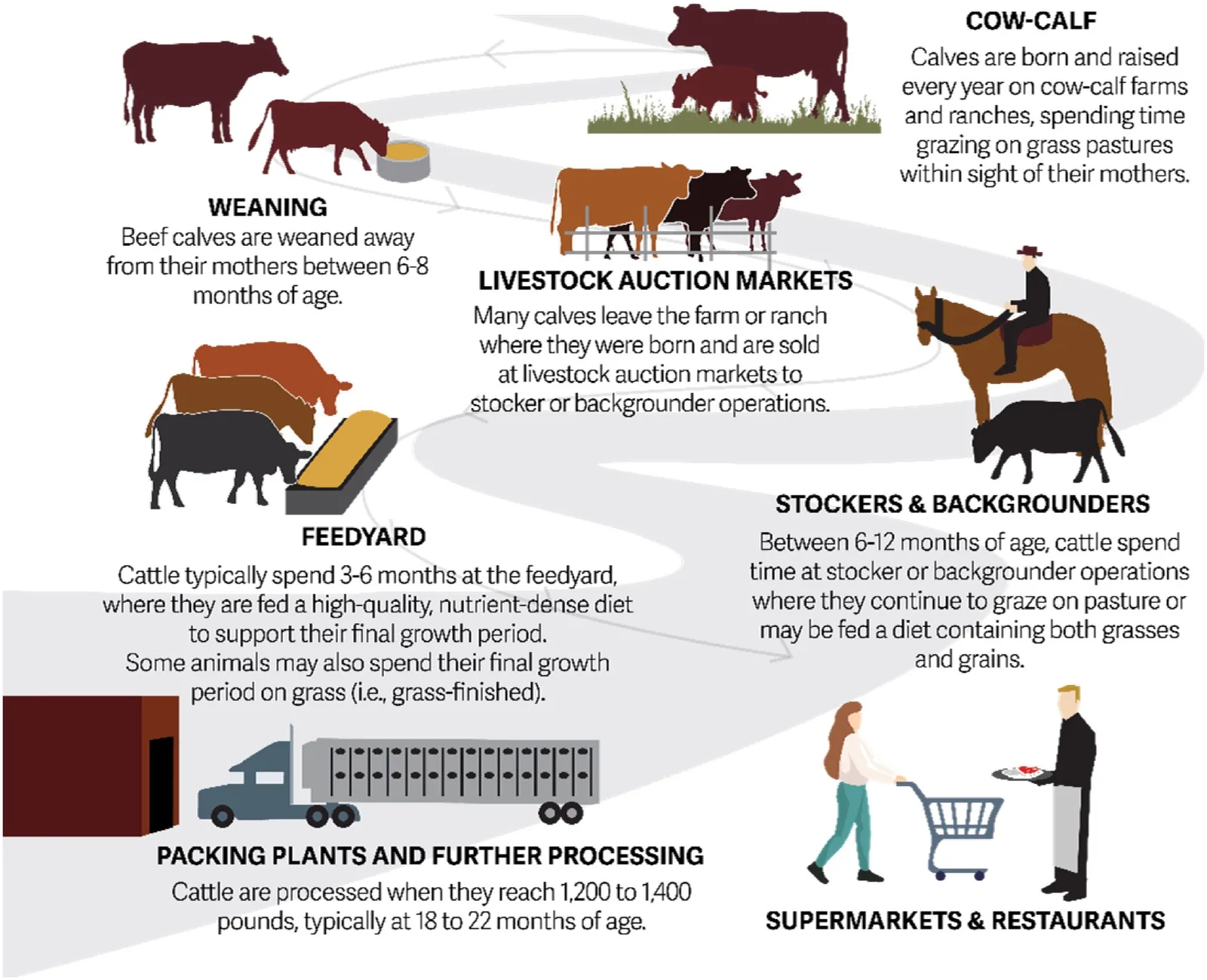
Conceptual overview of the U.S. beef supply chain, from cow-calf production through end-consumer. The schematic was developed by the authors based on published descriptions of U.S. beef production systems and commonly used life cycle assessment system boundaries, and is intended to provide contextual orientation for the complexities of the supply chain rather than a quantitative representation of flows or impacts.

To guide action, stakeholders across the U.S. beef value chain have pursued greater alignment on where research can most effectively advance environmental outcomes. In 2022, the U.S. Roundtable for Sustainable Beef (USRSB) established industry-wide sustainability goals and sector-specific targets across six high-priority indicators of sustainability, providing a unifying framework for consistent communication, coordination, and meaningful action (USRSB, 2022). These goals are the result of multistakeholder collaboration and were set to provide a north star for industry action, while sector targets were set to: 1) demonstrate where each sector can take ownership of its actions to support advancement toward the overarching goals and 2) how other sectors can support one another, based on these prioritized areas of focus.

Given the pressure placed on the beef supply chain to reduce emissions, the USRSB goal areas of Air & Greenhouse Gas Emissions, Land Resources, and Water Resources are the areas where clarity and alignment on action are needed most urgently (Lai et al., 2026). These areas combine high potential for measurable improvement with substantial remaining scientific uncertainty and growing importance to downstream supply-chain partners working to meet their own sustainability commitments. Furthermore, the live-animal stages of production exert the greatest influence on environmental outcomes and are where the most significant knowledge gaps persist and prevent meaningful progress. Figure 2 provides an overview of the industry-wide goals and Cow-Calf (including stocker) and Feedyard (including backgrounder) sector targets set by USRSB across the indicator areas of Air, Land, and Water.

**Figure 2.**
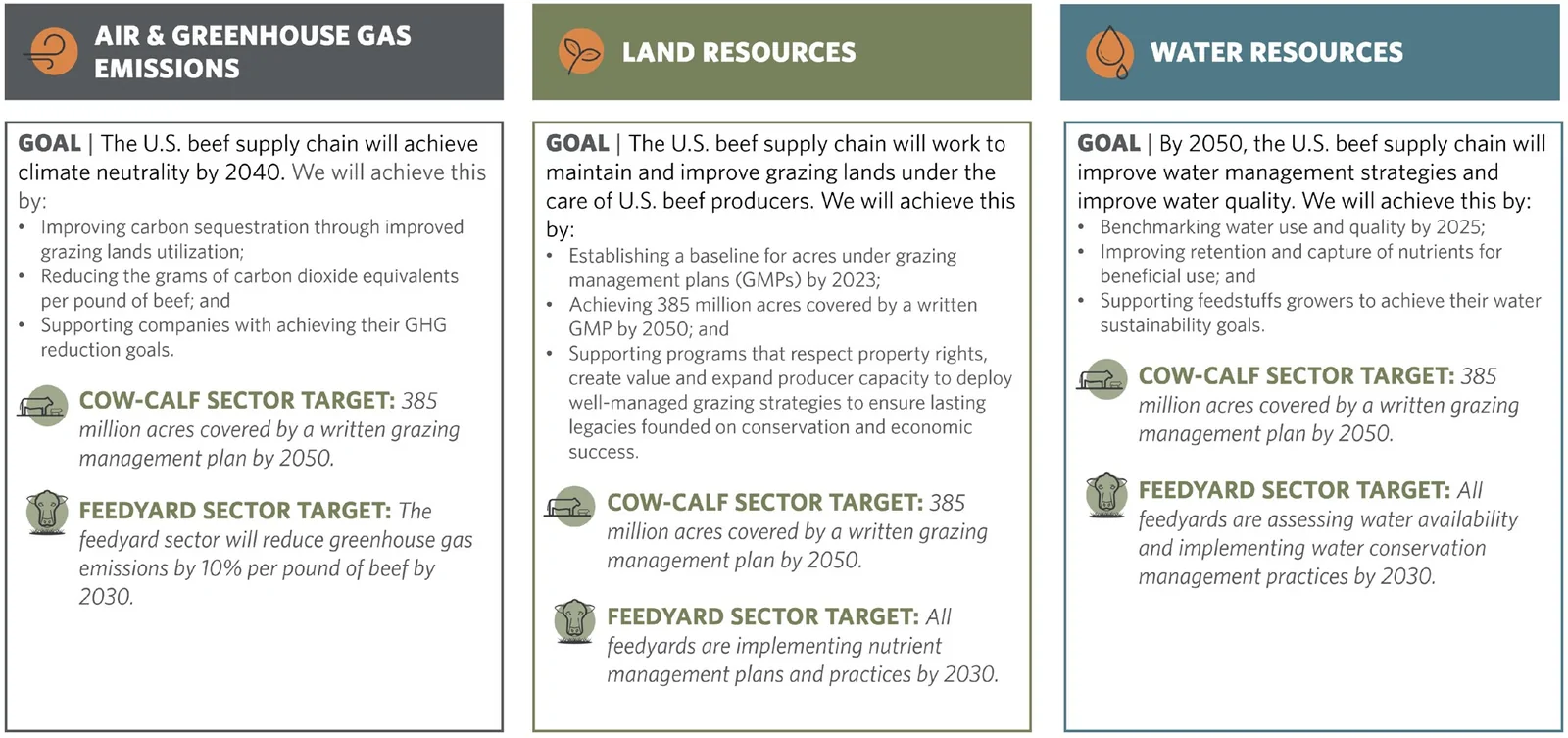
U.S. Roundtable for Sustainable Beef’s industry-wide sustainability goals for the three environmental indicator areas of air and greenhouse gas emission, land resources, and water resources, and corresponding targets of focus for the cow-calf and feedyard sectors (USRSB, 2022).

In order to support continued alignment and coordinated action, there is a pressing need for a coordinated, cross-sector research agenda. The work herein aims to bring such an agenda to life through the creation of a research roadmap that is anchored in industry-defined priorities, supports consistent measurement, and directs investment to the highest-impact opportunities.

Organized within the USRSB environmental goals framework, this roadmap aims to clarify where improvement potential is greatest and where stronger evidence is most urgently needed across the U.S. beef supply chain. It offers a clear, investable research agenda spanning measurement, technology, modeling, genetics, and producer practicality and is designed to support continuous improvement, strengthen data credibility, and accelerate progress. In short, this work moves the conversation from why alignment is needed to what to do next, prioritizing research that producers can adopt, supply chains can verify, and the industry can scale.

## Roadmap Methodology

While Lai et al. (2026) make the case for a coordinated, cross-sector research agenda, highlighting fragmentation, inconsistent methodologies, and limited alignment that currently constrain progress, the work herein advances the next step: a practical, science-based roadmap that identifies the critical research needs required for sustained, credible improvement across the U.S. beef value chain.

The research priorities presented herein were developed through a structured, multistep process that combined literature synthesis, subject matter expert (SME) leadership, and broad, multistakeholder engagement to build consensus. This process was designed to ensure that the resulting roadmap reflects both the current state of scientific knowledge and the practical considerations necessary for implementation across diverse beef production systems.

USRSB partnered with the Foundation for Food & Agriculture Research (FFAR) to convene academic and industry experts through a series of virtual focus group sessions followed by an in-person workshop. These engagements were designed to identify key knowledge gaps, evaluate emerging areas of research, and refine priorities through facilitated discussion and iterative feedback.

### Subject matter expert leadership

Eight subject matter experts (SME) were selected to guide information gathering and support development of the research roadmap. SMEs were chosen based on their demonstrated expertise in beef production systems and environmental sustainability and were responsible for synthesizing available scientific literature, identifying key knowledge gaps, and facilitating technical discussions. SMEs were organized across the three primary environmental indicator areas as follows:

- Air & Greenhouse Gas Emissions
  - Dr. Sara E. Place, Colorado State University; Dr. Troy Rowan, University of Tennessee Knoxville; Dr. Logan Thompson, Kansas State University
- Land Resources
  - Dr. Paige Stanley, Colorado State University; Dr. Jeff Goodwin, Texas A&M Center for Grazinglands and Ranch Management; Dr. Jason Rowntree, Michigan State University
- Water Resources
  - Dr. Kevin Wagner, Oklahoma State University; Dr. Susan Metzger, Kansas State University

Across these indicator areas, SMEs synthesized current scientific understanding and developed initial draft research priorities that informed subsequent stakeholder engagement activities. The in-depth reviews that supported the workshop activities and subsequent development of the roadmap herein are available in the companion articles: Thompson et al. (2026), Stanley et al. (In press), and Wagner et al. (In press).

### Focus group sessions

USRSB, FFAR, and a third-party facilitation team hosted three publicly accessible virtual focus group sessions corresponding to the three environmental indicator areas: Air & Greenhouse Gas Emissions, Land Resources, and Water Resources. Each session was led by SMEs and designed to assess the current state of the science, identify promising research directions, and highlight key gaps requiring further investigation.

Each session included participation from a broad cross-section of stakeholders across the beef value chain, including producers, packers and processors, retailers and food service companies, nongovernmental organizations, researchers, and other industry partners, with more than 80 participants engaged per session. These discussions provided critical input to ensure that the emerging research priorities reflected diverse perspectives and real-world considerations. Feedback gathered during these sessions was used to refine and expand draft research topics and identify additional areas of importance for further evaluation.

### In-person workshop

Following the virtual engagement process, USRSB and FFAR convened a 2-d, facilitated, in-person workshop to further refine and prioritize the research roadmap. Eighty participants representing more than 50 organizations across the beef value chain participated, including again producers, packers and processors, retailers and food service companies, nongovernmental organizations, researchers, and other industry partners.

The workshop built upon draft research priorities developed through SME analyses and focus group input, including synthesis of current scientific knowledge presented in companion topic areas addressing air, land, and water systems. The workshop began with expert panel presentations summarizing the current state of the science and initial identification of research gaps, followed by a plenary discussion to clarify key issues and gather additional input from participants.

The majority of the workshop was dedicated to structured breakout sessions, where participants reviewed and provided feedback on draft research priorities and worked collaboratively to refine, modify, and prioritize research topics. This iterative process enabled integration of scientific expertise with stakeholder perspectives, resulting in a consensus-driven set of research priorities that reflect both scientific rigor and practical applicability.

## Research Agenda

Accurate, scalable, and cost-effective measurement methods are essential to meet the research needs across the environmental indicator areas of Air & Greenhouse Gas Emissions, Land Resources, and Water Resources. There was a consensus from the extensive stakeholder input gathered to develop this roadmap that to fully address the industry’s needs for sustainable advances, long-term, coordinated, and interdisciplinary research efforts are essential. Greater quality and quantity of data are clearly needed to increase understanding of the key metrics that should be used across research efforts to support more accurate assessments and effective information sharing. Major barriers that exist around data and data sharing include ownership, control, and privacy. Inherent in efforts to support addressing research needs will be a need to recognize and work to resolve these sorts of barriers through clear data governance and an enabling and trusting environment when working alongside producers.

Importantly, decisions intended to improve one sustainability outcome may introduce trade-offs or unintended consequences across other environmental, economic, or social dimensions. For example, practices aimed at reducing greenhouse gas emissions may influence water use, land productivity, input costs, or management complexity, with variable implications depending on region and production system. Consequently, no single practice or technology should be assumed to universally optimize outcomes across air, land, and water. This research roadmap, therefore, emphasizes systems‑level evaluation and producer practicality, recognizing that progress toward sustainability requires careful consideration of synergies, trade-offs, and context‑specific impacts rather than one‑dimensional solutions.

Supporting the research needs outlined in this roadmap will ultimately drive adoption of best management practices across the cow-calf and feedyard stages of the U.S. beef value chain, providing opportunities for producers, scientists, and supply chains to optimize U.S. cattle production for environmental outcomes. Figure 3 provides a high-level summary of the sustainable beef research roadmap, featuring the identified critical research areas, corresponding environmental sustainability indicator areas, and key research topics identified. In total, six critical research areas and 22 key research topics across the three sustainability indicator areas were identified. Further details for each priority research area and topic can be found in the subsequent sections below.

**Figure 3.**
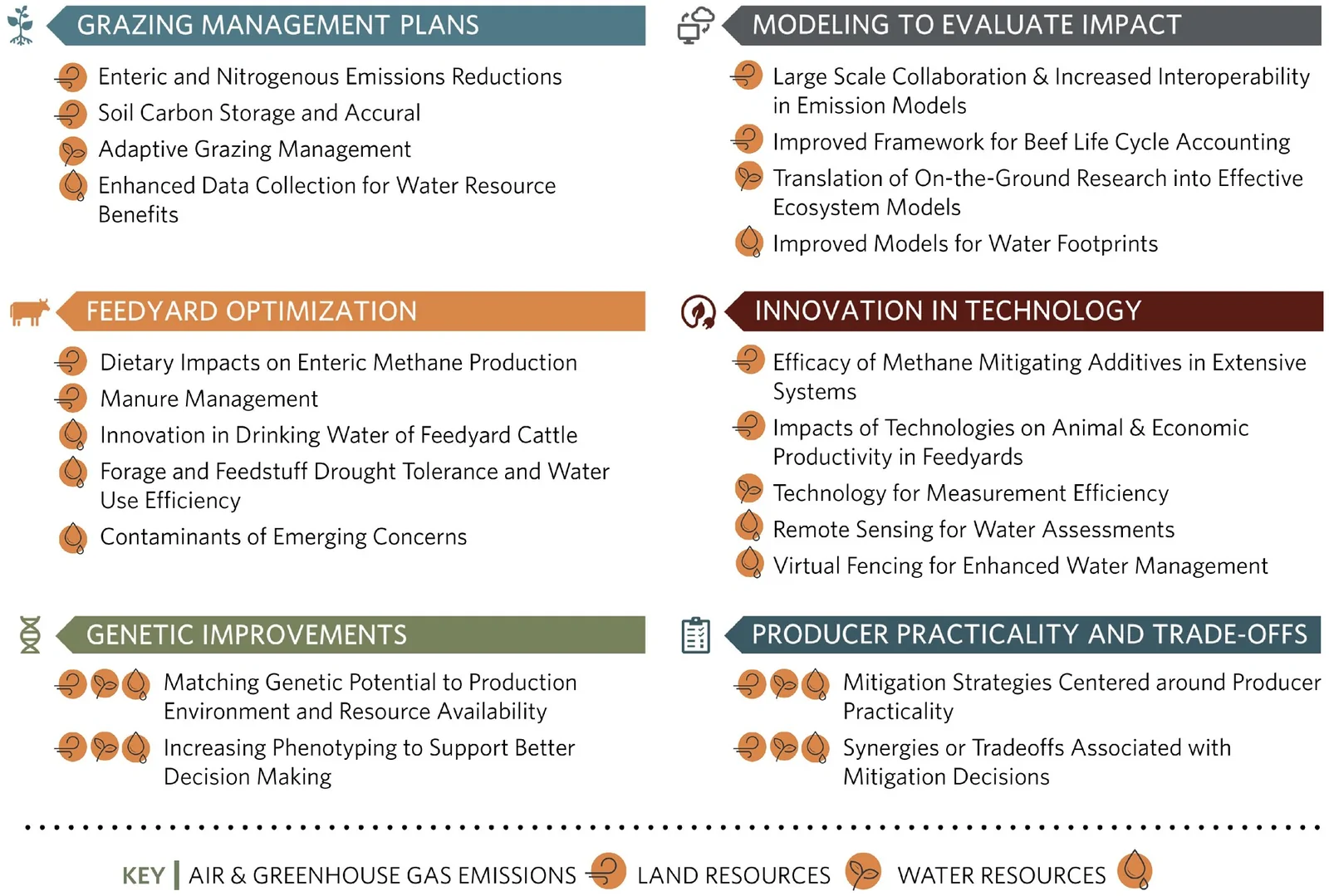
Sustainable beef research roadmap critical research areas and key research topics by corresponding environmental sustainability indicator areas of air and greenhouse gas emission (Place et al., 2026), land resources (Stanley et al., 2026), and water resources (Wagner et al., 2026).

**Figure 4.**
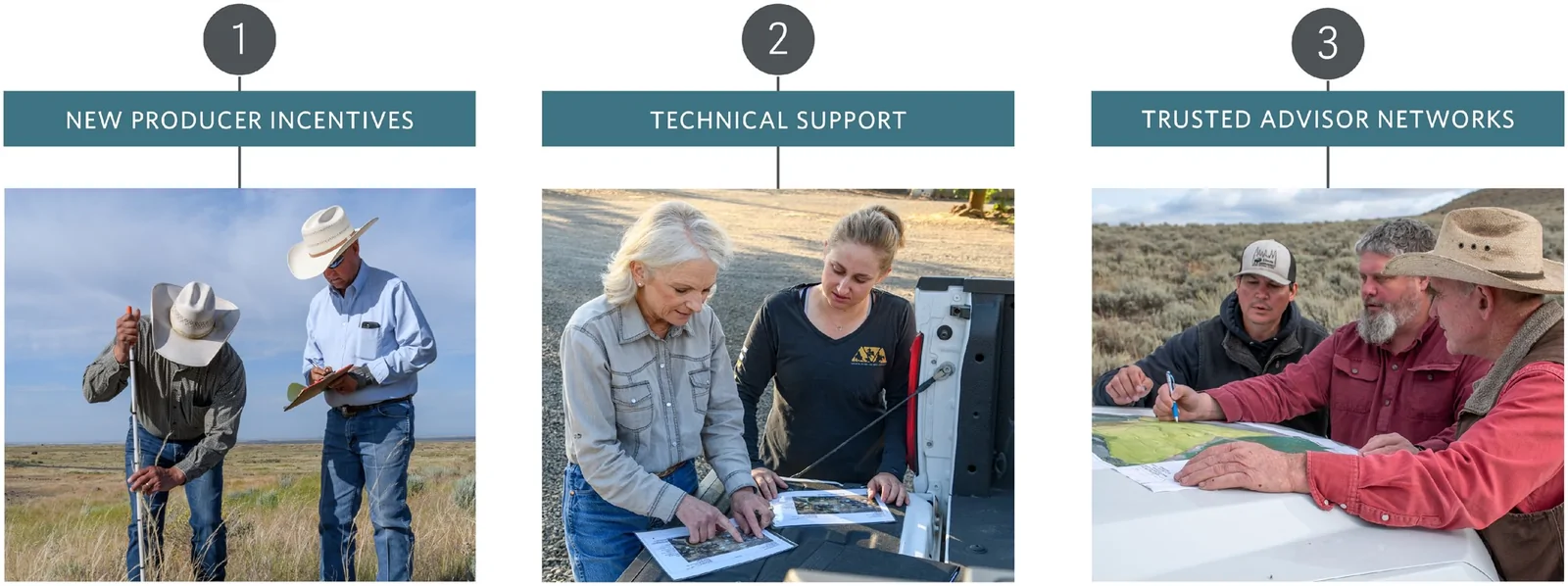
Critical areas of innovation and action for progress in the U.S. beef value chain.

### Critical research area: grazing management plans

Grazing cattle present a key opportunity to make meaningful progress toward the environmental indicator areas of Air & Greenhouse Gas Emissions, Land Resources, and Water Resources. For the cow-calf sector, implementation of a grazing management plan (GMP) is the primary metric to assess progress toward USRSB’s sustainability targets. Grazing management plans present a mechanism to enhance management capabilities across the cow-calf sector, bringing value to producers across the United States, while anecdotally providing the foundation for making progress toward industry environmental goals. To this end, the USRSB target aims to have 385 million acres operating under written GMPs by 2050. Details on how this ambitious target was set are available in (USRSB, 2022).

Having a GMP does not necessarily guarantee improved environmental outcomes; however, the act of writing a plan down may support producers to perform a more critical assessment of their operation and develop a clearer understanding of actions that can be taken to achieve operational goals (Likins et al., 2024). The intent and focus of GMP uptake is to provide a tool that any land manager can implement. Meanwhile, the intent for addressing research needs tied to grazing management planning herein is to concretely demonstrate the value of written GMPs to support producer decision-making and operational goals, as well as determine the key elements and actions that can be used to demonstrate progress toward achieving industry environmental goals around air, land, and water. If successfully addressed, then further mechanisms for GMP uptake and means of measuring progress can be further defined.

#### Air and greenhouse gas emissions

Grazing cattle, or the cow-calf sector, represent just over 50% of the U.S. beef carbon footprint (in carbon dioxide equivalents, CO_2_e), with enteric methane (CH_4_) representing nearly 76% of the sector’s emissions (Rotz et al., 2019; Putman et al., 2023). Beyond enteric CH_4_ emissions from grazing animals, nitrogenous emissions, such as nitrous oxide (N_2_O) and ammonia (NH_3_), present other important sources of emissions from grazing animals (Rotz et al., 2019; Thompson and Rowntree, 2020). In addition to emissions from grazing animals, grazing lands play a key role in soil carbon storage and accrual, yet the magnitude remains uncertain due to a lack of robust research (Stanley et al., 2024; Watts et al., 2025). Soil carbon accrual presents an important opportunity to offset emissions from grazing cattle. With this in mind, two critical research areas for grazing cattle are outlined below.

##### Enteric and nitrogenous emissions reductions

1. How does the implementation of grazing management impact enteric and nitrogenous emissions?
  - What are the place-based considerations that enable GMPs to support best management practices for emissions reductions?
  - What are the key areas of focus within a GMP to support quantifiable emissions reductions (e.g., forage quality, soil moisture, ground cover, etc.)?
2. What technologies exist to support more reliable, real-time, and efficient measurement of enteric emissions in grazing systems?
3. What tools and/or best management practices provide practical, scalable, and economically feasible enteric methane reduction opportunities?

##### Soil carbon storage and accrual

1. How does the implementation of a GMP impact soil carbon storage and accrual?
  - What are the place-based considerations that enable GMPs to determine best management practices for soil carbon accrual?
  - What are the mechanisms that can link soil carbon to other important ecosystem services, including biodiversity, water retention and cycling, productivity, and resilience of both animals and the land?
  - Robust field measurements, scalable verification methods, and refined carbon accounting models are required to properly address grazing impacts on soil carbon.

#### Land resources

Well-managed grazing lands provide both ecological and economic benefits by improving soil health, increasing productivity, and enhancing resilience to climate change (Sollenberger et al., 2020; Rouquette et al., 2023). Adaptive grazing can aid in restoring functionality and productivity of degraded grazing lands, strengthen water retention, and support biodiversity while maintaining or even increasing productivity (Barnes and Howell, 2013; Grissom and Steffens, 2013; Likins et al., 2024). While substantial research has been conducted around adaptive grazing, important gaps remain in scaling findings across diverse production systems and translating results into operationally feasible practices. Furthermore, many grazing lands are unsuitable for cropping and can more easily enhance soil carbon storage and accrual through strategic grazing, but the extent of carbon sequestration potential across different regions and grazing systems is uncertain (Stanley et al., 2024). Grazing management plans help producers optimize resources and plan more strategically and adaptively, yet their impact on ecosystem services remains unclear (Díaz-Pereira et al., 2020). Understanding the relationship between these factors is essential for guiding conservation and production strategies that maximize environmental and economic benefits over time. With this in mind, one critical research area for grazing cattle is outlined below.

##### Adaptive grazing management

1. How do adaptive grazing levers (e.g., frequency, timing, intensity, and duration) quantifiably affect grazing land ecosystem services, such as soil carbon, biodiversity, and resilience?
  - A combination of controlled experiments paired with measurements on working ranches, long-term monitoring, and both wide-scale and region-specific studies is necessary to address this topic effectively.
2. What are the best strategies for restoring functionality and productivity to degraded grazing lands?
  - How do GMPs and their implementation support quantifiable improvements?
3. What are the economic benefits associated with GMPs and the synergies between soil health, biodiversity, and livestock performance?

#### Water resources

Water use and water quality in grazing cattle are complex topics, and the use of GMPs to support improvements in both water use and quality is not well studied. Grazing cattle account for around 59% of blue water use (i.e., water sourced from surface and groundwater reservoirs) across the live animal stages of beef production (i.e., cow-calf through feedyard), and the primary source of blue water use is irrigation for feed production (Rotz et al., 2019; Klopatek and Oltjen, 2022). In regard to water quality, grazing cattle contribute to around 63% of nitrogen, primarily in the form of nitrate (NO_3_^−^) emissions leaching to groundwater across the live animal stages of beef production (Rotz et al., 2019). Both water use and quality, however, are highly variable by region of production. Some studies have demonstrated the ability of well-managed grazing to support better water retention and holding capacity in soils and filtering of excess nutrients in water run-off (Wagner et al., 2012; Abdalla et al.,2018; Kim et al., 2023). While some data provide evidence that well-managed grazing strategies support improvements in use and quality, further research is necessary to best understand how GMPs support these improvements. With this in mind, the following critical research area for grazing cattle is outlined below.

##### Enhanced data collection for water resource benefits

1. How do GMPs support quantifiable effects on water quality and other key water and productivity indicators (e.g., soil health, soil moisture, water infiltration, drought resilience, etc.) across a variety of production settings?
  - Long-term, ranch- and watershed-scale studies across a variety of environments are necessary to effectively address this question.
2. How can novel technologies, such as continuous measurement sensors, support measurement of water quality, runoff, stream flows, soil moisture, vegetation, cattle location, etc., to better understand the role GMPs might play in supporting improvements in water use and quality?
  - Is there an opportunity to combine some or all of these technologies to achieve water use and water quality goals?

### Critical research area: feedyard optimization

#### Air and greenhouse gas emissions

The uniqueness of feedlot settings creates a variety of emissions in the U.S. beef system. However, many of these emissions are within the control of feedyard management through a variety of strategies (Hristov et al., 2013; Waldrip et al., 2015; Beauchemin et al., 2020). There is a linkage between what an animal eats and how that interacts with enteric methane production while in confinement. While substantial research has been conducted in the area of mitigating emissions from feeding cattle, important gaps remain in understanding the co-supplementation impacts of various amendments, as well as scaling findings across diverse production systems and translating results into operationally feasible practices. A greater understanding of how feedyard diets impact enteric methane emissions provides an opportunity for more effective decision-making at the feedyard level. In addition to GHG emissions, volatile organic compounds (VOCs) and reactive nitrogen (N) are important emissions to consider at the feedyard level (Waldrip et al., 2015). With this in mind, key research needs for emissions reductions in the feedyard sector include the following.

##### Dietary impacts on enteric methane production

1. What are the comprehensive dietary impacts on enteric methane emissions from feedyard cattle, and where are the strongest direct correlations observed?
2. How do feed inputs, supplemental fats, tannins, and grain processing impact enteric emissions, and what are the interactions with additives such as ionophores and methane inhibitors?

##### Manure management

1. What are the best means of storing feedyard manure to best control for VOCs and reactive N?
  - How might storage need to be addressed in different feedyard systems (e.g., enclosed barns versus dry lots)?
2. What compounds result from feedyard manure, and what environmental and management conditions lead to the development of reactive N?
3. What are the best methods for the use of measurement technologies, and what are practical mitigation options?

#### Water resources

Water use, availability, accessibility, and quality are among the most crucial factors in cattle production. Regardless of operation size, ensuring access to high-quality drinking water for animals is essential, along with implementing management practices to minimize waste. Wastewater from lagoons provides an opportunity to rethink recycling water for cattle consumption as a means of water conservation. Along with minimizing waste, water use in forage and feedstuff crops is a key consideration, as crops purchased for feed are one of the key contributors to feedyard blue water use. Alternative forages and grains, such as sorghum, millet, and triticale, can contribute to water conservation in cattle production by reducing overall water consumption, improving soil moisture retention, and enhancing drought resilience; however, lagging prices and research gaps lead to a preference for traditional grains fed to cattle. Water quality is another important consideration in relation to the feedyard sector and waste management, and treatment is an important consideration for both environmental and public health. Specifically, more information is needed to fully understand contaminants of emerging concern (CEC) and the ways to minimize impacts to water quality from lagoons and wastewater. With all of this in mind, the following are key research areas for water resources in the feedyard sector.

##### Innovation in drinking water for feedyard cattle

1. What are acceptable drinking water standards for feedyard cattle, and can innovative applications for lagoon wastewater treatment provide a means of recycling water in feedyards?
  - How does drinking water quality impact cattle health, performance, and meat quality?
  - What are the most economical technologies for treating water to levels safe for cattle consumption?

##### Forage and feedstuff drought tolerance and water use efficiency

1. Are there opportunities for innovation with drought-tolerant and low-water-use feedstuff alternatives to support similar or better animal productivity while reducing water requirements for cattle feed?
  - Innovation might include plant genetics, precision farming, or optimizing processing techniques, digestibility, and nutrient compositions.
  - Considerations of the location of crop production and opportunities based on aquifer and water table needs of the region should be at the top of mind.
  - Tradeoffs between water efficiency and animal performance and resulting enteric emissions must also be considered.

##### Contaminants of emerging concern

1. How do pharmaceuticals, per- and polyfluoroalkyl substances (PFAS), and other CECs move, transform, and respond to treatment within different components of animal waste management systems, and what are the implications for environmental and public health?
2. How can waste management systems be modified to enhance the treatment and removal of CECs?

### Critical research area: innovation in technology

#### Air and greenhouse gas emissions

Advances in technology have been seen across a multitude of agricultural applications. In the case of emissions measurement and mitigation from cattle, several tools have been developed, including sniffers, head gear, water amendments, slow-release boluses, and a variety of feed additives, which continue to be developed (Tedeschi et al., 2022; Ungerfeld et al., 2022). It is critical that researchers conduct experiments that focus on total carbon, nitrogen, and energy balance experiments with feed additives, while also attending to safety and efficacy (Beauchemin et al., 2020). Application opportunities and research needs are variable depending on the phase of production; however, there are two critical areas of focus for research needs.

##### Efficacy of methane mitigating additives in extensive systems

1. What slow-release mitigation options are viable tools for reducing emissions across production systems?
2. What is needed to enhance the mechanistic understanding of the mode of action for methane inhibitors?
3. How do variable diets impact the efficacy of feed additives or novel technologies?
4. What opportunities exist for early life interventions (e.g., nutritional, microbial, or genetic strategies applied during early development to influence lifetime emissions or productivity)?

##### Impacts of technologies on both animal and economic productivity in feedyards

1. How do feed additives or novel technologies impact animal productivity, and what are the full life cycle impacts when assessed under large-scale feeding experiments?
  - What are the impacts of producing and distributing novel additives or technologies in relation to the mitigation opportunities from the product?
2. What are the practical feeding applications for methane inhibitors and the corresponding effectiveness across representative diets, implications on productivity, and impacts on farm economics?
3. What implications do feed additives or novel technologies have on input costs, and what opportunities exist to ensure economic viability?

#### Land resources

Current methods for measuring grazing impacts on soil and ecosystem services are slow, costly, and difficult to scale. Advancing new measurement technologies and integrating producer data can improve research outcomes. Process-based models, remote sensing, and on-the-ground verification are critical for bridging data gaps and improving accuracy. With this in mind, there is one key research need related to land resources.

##### Technology for measurement efficiency

1. What technologies can improve measurement accuracy and scalability, and are available to bridge data gaps and improve accuracy for assessment of GMPs and impacts to soil carbon, biodiversity, and resilience?
  - Integration of producer data provides an opportunity to enhance measurements from process-based models, remote sensing, and on-the-ground verification.

#### Water resources

Satellite imagery and remote sensing are powerful tools for tracking water use, availability, and efficiency in livestock operations. By using various remote sensing technologies, producers and researchers can assess water conditions over large areas, reducing reliance on manual monitoring and improving resource management. In addition to opportunities with remote sensing, virtual fencing is a relatively new technology that shows promise for enhanced water management in extensive systems. This tool allows producers to implement virtual fences and manage grazing, including moving cattle to and from areas or restricting animals from certain areas, without a physical fence in place. Ongoing research efforts around the potential benefits to water quality and riparian management using virtual fencing are currently being studied at scale; however, more work is needed to fully understand the potential abilities of this technology. With these technologies in mind, key research opportunities around Water Resources include the following.

##### Remote sensing for water assessments

1. What opportunities exist to leverage on-the-ground technology to support more reliable remote sensing?
2. Additional research is needed to improve the reliability of remote sensing by integrating satellite data with on-the-ground technology, while reducing costs and addressing concerns of data security and privacy.

##### Virtual fencing for enhanced water management

1. How effective is virtual fencing at supporting water management and water quality goals, at scale, on grazing operations?
2. What are the key advances needed across virtual fencing technologies to support producer adoption, ease of use, and economic viability on the farm?

### Critical research area: modeling to evaluate impact

Across air, land, and water, modeling is an essential tool to support benchmarking critical metrics across beef production and evaluating progress made across the beef industry over time. Several lifecycle assessment models have been performed to better understand environmental impacts across the beef industry, and these models support identifying hot spots where the industry has opportunity for advancement (Battagliese et al., 2013, 2015; Thoma et al., 2017; Tichenor et al., 2017; Asem-Hiablie et al., 2019; Rotz et al., 2019; Putman et al., 2023). Further work is necessary across air, land, and water models to help refine models and support effective decision-making at the operational level. Across all three indicator areas, opportunities exist for greater collaboration across research institutions and industry partnerships to promote more streamlined data collection that supports better-informed models and, by extension, more informed decision-making opportunities for producers and supply chain partners.

#### Air and greenhouse gas emissions

Comprehensive GHG emission measurements and updating current models to improve accuracy will allow the U.S. cattle industry to better characterize its baseline emissions and accurately quantify emissions reductions (Tedeschi et al., 2022). A target for emissions reductions cannot be set without first fully quantifying the baseline for GHG emission production. Updated models must be affordable, accurate, and utilize precise measurement technologies that scale from individual animal trials to whole operation assessments for monitoring, reporting, and verification (MRV) applications. A more mechanistic approach to modeling emissions, particularly enteric emissions, must be taken. Biological systems such as the rumen do not function linearly, and mechanistic models to better represent this system are needed. Furthermore, there is a pressing need for a framework that supports accurate assessment of emissions, accounting for the complexities of working in a biological system. To accomplish this, the scientific community will need to work cohesively in developing and testing new measurement technologies, proxy evaluations, frameworks, and models (Tricarico et al., 2022). Building national and/or regional consortia to share data and results and discuss experimental challenges may enable more timely advances in addressing modeling needs. Below is an outline of the key needs for improved and more relevant emissions models.

##### Large scale collaboration and increased interoperability in emissions models

1. What affordable, scalable, and accurate measurements can be conducted to inform more representative models?
  - How can a FAIR (Findable, Accessible, Interoperable, Reproducible) data approach be leveraged to ensure consistency across studies and support reliable models?
2. How can data access pinch points be overcome to create comprehensive and regionally representative data sets to update baseline data for the industry?
3. What opportunities exist for streamlined data sharing across universities, as well as anonymized data from industry partners, to support pooling data in a centralized location, enable building more informative data sets, and support model validation over time?

##### Improved framework for beef life cycle emissions accounting

1. What is needed to make scalable, accurate, and affordable models a more useful mechanism for emissions accounting by supply chain actors?
2. How can modeling better enable ranchers and landowners to make decisions for best management practices while supporting more accurate emissions accounting of biological systems needed for supply chains?
  - What opportunities exist to inform a new market based on these actions?

#### Land resources

Limitations and discrepancies from prior research on grazing, soil, and ecosystem outcomes from grazing cattle create major hurdles for effectively modeling land resources. Effective models for grazing cattle need to incorporate trade-off evaluation of outcomes related to soil, water, biodiversity, productivity, and producer profitability. Such models can support not only producer decision-making and development of improved sustainability commitments but also science-based monitoring and communication around the effects of various grazing strategies. Key areas of interest for ecosystem models include the following.

##### Translation of on-the-ground research into effective ecosystem models

1. What are the key indicator areas necessary to assess during on-the-ground research that will support the development of accurate ecosystem models that provide insights into how grazing cattle affect soil, water, biodiversity, productivity, and producer profitability?
2. What technologies can support more consistent and streamlined data?
3. What opportunities exist to support collaboration across research groups to ensure interoperability of data and strengthening of ecosystem models?

#### Water resources

Watershed-scale data gaps on water quality, soils, and other natural resources need to be addressed to help improve models and further understand changes needed in grazing to support producer and ecosystem benefits. Through the implementation of long-term, replicated watershed-scale field studies on water resource response to grazing, modeling improvements can be made, allowing improved predictions of water resource impacts under different hydrologic and grazing conditions. Furthermore, an opportunity to support decision-making and predict water outcomes on the farm arises with advances in technology like artificial intelligence (AI) and machine learning (ML). Hydrologic, water quality, and water footprint modeling stand to benefit from the availability of new continuous data streams and the use of AI and ML approaches. Specifically, the following needs to be prioritized to address water footprinting needs.

##### Improved models for water footprints

1. What tools can effectively simulate the effects of GMPs on water resources and water quality?
  - Improved simulation tools must incorporate spatial variability (e.g., soil and vegetation characteristics) and a range of management alternatives available to support water resource goals.
2. How do we better integrate hydrologic, water quality, and other production and ecosystem modeling with economic models to improve understanding of trade-offs of various grazing management strategies?

### Critical research area: genetic improvements

Genetic improvement is a long-term, permanent, and cumulative approach to mitigating air, land, and water impacts, particularly in grazing animals (Pickering et al., 2015). Genetic improvement is the product of using selection tools, such as expected progeny differences (EPDs) and selection indices, to identify animals with high genetic merit and deploy those genetics across the industry to drive improvements. Numerous examples of genetic selection driving improvements to production efficiency exist across the beef industry (Kuehn and Thallman, 2016; Rowan, 2022). At the heart of genetics and beef sustainability, one critical area of research is understanding maternal and terminal animal efficiency across diverse environments (Snelling et al., 2019). While genetic selection tools cover many profit and sustainability drivers, gaps exist, particularly in measuring efficiency traits in grazing contexts. Investments in phenotyping can support fine-tuning current selection tools for improved maternal and terminal efficiency as well as the development of novel genetic predictions (e.g., for methane emissions). Continued implementation of best practices like crossbreeding and using selection indexes will be essential for continued sustainability improvements. Key research areas where genetic improvement can support maximizing profitability while reducing environmental impact from cattle include the following.

#### Matching genetic potential to production environment and resource availability

1. For different environments and producer breeding goals, what existing selection tools would optimize maternal cow performance? What additional traits should be measured to help better inform these decisions?
2. In a feedyard setting, what traits are necessary to optimize terminal animal performance, and what are their relationships (complementary or agnostic) with efficiencies in the cow-calf sector?

#### Increasing phenotyping to support better decision making

1. What tools are needed to support producers in collecting sufficient phenotypes (e.g., body weights, feed efficiency, methane phenotypes, etc.) to enable informed selection and mating decisions for cow-calf producers?
2. What technology and/or communication mechanisms are needed to transfer phenotypes collected at either feedyards or packers back to cow-calf producers to support EPD development, decision support tools, and expanded marketing opportunities?

### Critical research area: producer practicality and trade-offs

Research with producer involvement and practicality is imperative to not only ensure successful solutions, at scale, but also create a mechanism for producer feedback, generate buy-in, and, ultimately, encourage widespread, sustained producer adoption of evidence-based mitigation strategies. Examining cross-cutting connections between air, land, and water is essential as none of these topics is isolated, and research that comprehensively examines synergies and trade-offs simultaneously will be most effective. The analysis of producer practicality, synergies, and trade-offs across air, land, and water must be at the forefront of all future research if it is to succeed in supporting continuous improvement across beef production. With producer practicality and assessment of trade-offs in mind, below are key research opportunities.

#### Mitigation strategies centered around producer practicality

1. What mechanisms support designing research alongside producers, with practicality, viability, efficacy, and profitability in mind?
2. How can researchers be supported to share knowledge across producer networks so that practical applications can be implemented across a multitude of operations and operation sizes?

#### Synergies or trade-offs associated with mitigation decisions

1. What are the synergies or trade-offs associated with decisions made on mitigating for one environmental factor over another, and how do these decisions further impact full-picture sustainability of operations (e.g., animal well-being, animal efficiencies, economic viability, etc.)?
2. How might life cycle assessment models be more effectively utilized to understand synergies and trade-offs associated with mitigation decisions and support more effective communications with producers on the practicality of different decisions or best management practices?

## Critical Areas of Innovation and Action

Advancing sustainability in the cattle industry requires a multifaceted approach that addresses the social, economic, and environmental outcomes of beef production. Critical areas of innovation and action must be centered around supporting producers on the ground (Figure 4).

### Producer incentives

In some global contexts, sustainability outcomes are driven in part by compliance-based or punitive policy mechanisms. While such approaches may accelerate adoption in certain systems, their applicability within the diverse and highly heterogeneous U.S. beef sector remains uncertain. Incentives that encourage producers to adopt sustainable practices without compromising, and ideally enhancing, their profitability are a key area for innovation and action. These incentives can range from financial support to market access, helping bridge the gap between traditional practices and other innovative solutions. Equally important is strong advocacy for implementing these solutions, which fosters a supportive environment for change and highlights the benefits of sustainability for both producers and consumers. Ideally, the coupling of these two should be considered when encouraging the adoption of practices. As a result, producer support is a key target area for downstream supply chain partners to engage in more impactful ways (USRSB, 2022). Combined with collaborative efforts across the supply chain to support research needs identified herein, the opportunities for innovation and action can be amplified.

### Technical support

Ensuring producer access to accurate information, resources, and ongoing support has the opportunity to empower producers to make informed decisions about adopting sustainable practices and management strategies. Producers are more likely to embrace change when they can clearly see the value proposition, both in terms of profitability and environmental stewardship. Access to technical assistance further ensures that producers can successfully implement new practices and technologies, especially when they determine that these changes offer tangible benefits to their operations. This research roadmap and the goals put forward by USRSB have the potential solutions to help close the gap in technical assistance and scientific discovery to help propel producers forward. The pressing need for technical support highlights a critical area for industry-wide collaboration to fund people and programs.

### Trusted advisor networks

For decades, U.S. Extension agents and educators at land-grant universities have worked alongside producers and industry to help share and translate relevant university research. Strengthening the network of trusted technical advisors ensures that producers receive reliable, science-based guidance tailored to their specific needs. Expanding this advisor network is critical to meeting the growing demand for expertise in sustainable agriculture and fostering meaningful connections between research institutions and the farming and ranching communities. The research projects defined by this group of experts can help make this work possible.

## Conclusion

The U.S. beef supply chain has prioritized sustainable production for decades and must remain steadfast in actively supporting future sustainability efforts. From processors through retailers and researchers to other partners linked to the beef supply chain, every stakeholder has a role in promoting responsible practices, creating market incentives, and ensuring that the entire value chain is aligned with supporting the critical research areas and key topics addressed with this roadmap. While this roadmap is grounded in science and producer practicality, its implementation will occur within evolving policy and market contexts. Regulatory frameworks, environmental reporting requirements, and incentive structures, whether voluntary or mandatory, may influence which research pathways are prioritized and how outcomes are operationalized. As such, this roadmap should be viewed as adaptive, with periodic reassessment needed to ensure alignment with policy developments and regional realities. Importantly, realizing the full value of this research roadmap will require parallel attention to data governance, trade-offs, policy context, and evolving incentive structures alongside scientific advancement.

By working collaboratively across sectors and providing producers with the tools, knowledge, and support they need, the U.S. cattle industry can make significant strides toward a continued and sustainable future. This roadmap serves as a testament to the power that collaboration has and how it can advance science for generations to come. This roadmap should be looked at with high regard by individuals with the ability and interest in supporting science-based advances in sustainable beef production.

## Author Biographies

**Dr. Samantha J. Werth** serves as Executive Director with the Global Enteric Methane Impact Alliance (GEMIA). In this role, Sam works collaboratively alongside a variety of stakeholders to support advancement of applied solutions to reducing emissions from livestock production. Prior to her role with GEMIA, Sam served as Executive Director with the U.S. Roundtable for Sustainable Beef (USRSB) where she focused on building collaborative partnerships to support USRSB’s sustainability initiatives and supporting members with their sustainability efforts. Sam is a native of California and completed her undergraduate, master’s, and doctoral studies at the University of California, Davis. Her graduate studies centered around ruminant nutrition and sustainable beef production. Sam is passionate about leveraging science-based information to support advances in sustainable beef production.

**Grace D. Vehige serves** as the Communications Director for the U.S. Roundtable for Sustainable Beef and is the owner of 93 Ink, where she provides strategic communications related to storytelling, sustainability, digital media, grant development, and stakeholder outreach. Her experience spans the industry, including a current communications role with the Global Roundtable for Sustainable Beef and prior experience with the National Cattlemen’s Beef Association and the University of Arkansas. Raised in a beef production family, she brings firsthand industry experience to her work and holds a Master of Science in agricultural communications from Texas Tech University.

**Dr. Sara Place** is an Associate Professor and expert in livestock systems sustainability with over a decade of experience in academia, industry associations, and private industry. She joined Colorado State University’s AgNext, a research collaborative focused on sustainable animal agriculture, in August 2022. Sara’s research focuses on enteric methane emissions measurement and mitigation from cattle. Prior to CSU AgNext, she was the chief sustainability officer for Elanco Animal Health, senior director for sustainable beef production research at the National Cattlemen’s Beef Association, and an assistant professor in sustainable beef cattle systems at Oklahoma State University. She received her PhD in Animal Biology from the University of California, Davis, and a BS in Animal Science from Cornell University. Sara is a native of upstate NY, where she grew up on a dairy farm.

**Logan R. Thompson**, PhD is an Assistant Professor, Sustainable Grazing Systems at Kansas State University. Logan grew up on a registered horned Hereford ranch in central Texas, sparking his passion for improving beef production sustainability by developing practical solutions for producers. He began his bachelor’s degree at Sam Houston State University and completed it at Texas Tech University in 2015. He earned a Master’s in beef sustainability at Oklahoma State University in 2017 and a PhD in ruminant nutrition at Michigan State University in 2021. Before joining Kansas State University in August 2022, he was a post-doctoral fellow at Colorado State University. His research focuses on measuring greenhouse gas emissions in grazing systems, the impact of grazing management on ecosystem function, and nutrient utilization across production systems. Logan’s wife, Rachel, is a Senior Scientist in Ingredient Solutions R&D at MGP Ingredients in Atchison, KS.

**Troy N. Rowan** is an Assistant Professor, Beef Cattle Genomics and State Extension Specialist at the University of Tennessee’s Department of Animal Science. Troy grew up in Southwest Iowa on a small seedstock Charolais operation. He joined the faculty at UT in January of 2021, where his research uses genomics to understand and predict economically important traits in beef cattle. He is particularly interested in cow efficiency, local adaptation, heterosis, and genetic approaches to increasing beef cattle sustainability. His work aims to deliver tangible solutions to U.S. beef cattle producers while answering fundamental biological questions. Troy’s extension work supports producers and industry organizations across Tennessee and the United States.

**Dr. Douglas J. Goodwin** serves as Research Associate Professor for Texas A&M AgriLife Research and Extension and as Director of the Center for Grazinglands and Ranch Management within the Department of Rangeland, Wildlife, and Fisheries Management. The Center focuses on providing applied, ranch-relevant research and education, prioritizing the ecological and economic resiliency of grazingland resources and ranching operations. Prior to Texas A&M, Dr. Goodwin served as Conservation Stewardship Lead and Senior Rangeland and Pasture Consultant for Noble Research Institute in Ardmore, Oklahoma. Dr. Goodwin has over 24 yr of experience working directly with ranchers and grazingland managers, implementing stewardship-focused management and providing innovative solutions to grazingland issues.

**Jason E. Rowntree, PhD** is Professor of Animal Science at Michigan State University. Holding the Charles Stewart Mott Distinguished Professorship for Sustainable Agriculture, Rowntree’s research focuses on identifying the metrics and management that reflect ecological improvement in grazingland and other agricultural systems. Rowntree has given presentations throughout the United States, Australia, Canada, Europe, Saudi Arabia, Africa, and New Zealand. Rowntree has led or been a co-investigator on $27.75 million in funding to conduct food system research. Jason is also the co-director of the Center for Regenerative Agriculture at Michigan State University. His work in beef sustainability was featured in the movie Sacred Cow, and he, too, has been highlighted in The Washington Post, New York Times, Forbes, and many other popular media publications.

**Paige L. Stanley, PhD** serves as Research Associate at Colorado State University. Stanley’s studies focus on how grazing management affects ecosystem and soil processes related to carbon sequestration and stabilization. With a B.S. in Biology and Economics (Georgia College), M.S. in Animal Science (Michigan State University), PhD in Environmental Science (UC Berkeley), and a Postdoc in Soil and Crop Science (Colorado State University), she brings an interdisciplinary approach combining soil biogeochemistry, rangeland ecology, rancher sociology, animal science, and plant ecophysiology. Her research focuses on soil carbon outcomes from grazing management, optimizing carbon measurement methods, understanding rancher adoption of adaptive grazing, and informing science-based policies for more resilient rangelands. Ranchers are central to Stanley’s work, ensuring it is equitable and applicable to social-ecological systems.

**Susan T. Metzger** serves as the Director of the Kansas Water Institute (KWI), Kansas State University and KCARE. Susan is the Director of Strategic Interdisciplinary Program Development for Kansas State University. Susan has broad experience representing Kansas State University and Kansas agriculture. Before her work at K-State, she was the Deputy Secretary of the Kansas Department of Agriculture, where she served the farmers, ranchers, and agribusinesses of Kansas and assisted in the development and delivery of tools to grow the state’s agricultural economy. Prior to that, she was the Chief of Planning and Policy for the Kansas Water Office, where she was the team leader in the development and implementation of the Long-Term Vision for the Future of Water Supply in Kansas.

**Dr. Kevin L. Wagner** serves as the Director of the Oklahoma NSF EPSCoR Program and Director of the Oklahoma Water Resources Center at Oklahoma State University. As Director of the Water Center, he provides leadership and administration of the Center’s water research, outreach, education, and grant programs; leads Center efforts to increase engagement with the water resources community across Oklahoma and the United States; and facilitates development of interdisciplinary teams to address high-priority water resources issues. As Director of the EPSCoR Program, he provides leadership and administration of a large multi-institutional and multi-disciplinary research team aimed at finding Socially Sustainable Solutions for Water, Carbon, and Infrastructure Resilience in Oklahoma.
